# A methodology to design a performance management system in preventive care

**DOI:** 10.1186/s12913-018-3837-8

**Published:** 2018-12-29

**Authors:** Federico Rotondo, Lucia Giovanelli, Nicoletta Fadda, Alberto Ezza

**Affiliations:** 1 Department of Humanities and Social Sciences, University of Sassari, Via Roma, 151, 07100 Sassari, SS Italy; 2 Department of Economics and Business, University of Sassari, Via Muroni, 25, 07100 Sassari, SS Italy

**Keywords:** Preventive care, Methodology, Performance measurement system, Key performance indicators, Participatory action research

## Abstract

**Background:**

Preventive care has gained increasing attention in health reforms around the world due to its ability to reduce the burden of disease and to save health costs. Nevertheless, there is a gap in terms of the development of reliable systems to measure and evaluate performance of preventive care in order to support decision-making and increase service outcomes. The aim of this study is to define a methodology for designing a performance management system (PMS) in order to effectively support the planning, control and evaluation of preventive care and to identify the factors that influence such a process.

**Methods:**

The methodology is based on the participatory action research approach, which implies collaboration between researchers and practitioners. The study was articulated in four phases and carried out in an Italian regional healthcare system that was undergoing a major reorganization process.

**Results:**

The findings provide insights into the peculiarities that affect preventive care and highlight two categories of critical factors: general issues regarding the process and specific issues regarding preventive care. The first category includes the importance of interactions between academics, physicians and policy-makers, the impact of workloads and red tape on employee involvement and the increased conservation mechanisms during periods of institutional change. The second category concerns the strong heterogeneity of preventive activities within health organizations, the huge amount of regulations and the incompleteness of information systems.

**Conclusion:**

The development of a PMS for preventive care can best be served by collaborative methods that involve academics, professionals and policy-makers, whose roles and responsibilities must be clearly defined, and by an improvement in transparency and communication within organizations in order to enhance the involvement of different professionals at appropriate times and in appropriate ways. Key recommendations that may improve the maintenance and use of information systems are proposed to policy-makers.

**Electronic supplementary material:**

The online version of this article (10.1186/s12913-018-3837-8) contains supplementary material, which is available to authorized users.

## Background

Today, the prevention of disease is a key consideration for public health policy-makers worldwide and public health programmes increasingly focus, as never before, on health promotion and disease prevention to minimize the burden of disease and the associated risk factors. The US Affordable Care Act of 2010 and the European Health Policy framework, ‘Health 2020’, are examples of the major role that prevention plays in the global health agenda. Other than the medical importance of developing population-based and individual-based interventions for both primary prevention and secondary prevention, such an approach has been seen as a key tool to cope with the lack of resources associated with the recent financial crisis and to enhance the economic viability of public healthcare systems [1]. It is not surprising that the World Health Organisation (WHO) identifies preventive and promotive health services as a key component of universal health coverage, “ensuring that all people have access to needed promotive, preventive, curative and rehabilitative health services, of sufficient quality to be effective while also ensuring that the use of these services does not expose the user to financial hardship”, which represents one of its major priorities [2].

In spite of the growing attention paid to the topic, the literature has mainly focused on the issue of equal access to preventive services [3, 4], while much less attention has been devoted to preventive care managerial practice. There is certainly a lack of empirically based analysis on the development, implementation and use of managerial systems in the field of prevention and, to date, no comprehensive and multidimensional framework to manage the performance of preventive services has been developed [5]. In addition, from a methodological point of view, whilst a number of studies have provided general recommendations to develop performance measurement systems in the healthcare sector and have highlighted the need to create a common language between and within countries [6–9], such a contribution has never been provided with reference to preventive care.

The aim of this research is to define a methodology for designing a performance management system (PMS) in order to effectively support the planning, control and evaluation of preventive care and to identify the factors that influence such a process. In particular, a participatory action research approach was used to design a PMS in an Italian regional healthcare system undergoing a major reorganization process.

## Developing performance measurement systems in healthcare and in preventive care

Several countries around the world have introduced PMSs to improve rationality in decision-making, thereby enhancing the overall performance of health systems and increasing accountability to taxpayers and stakeholders about the use of resources [10].

Among the main critical factors for the effectiveness of performance measurement, scholars have highlighted the multiple dimensions and constituencies surrounding the concept of health performance [11] and the difficulty of understanding the complex relationship between the outcomes of services and the efficiency of their delivery [12, 13]. Furthermore, PMSs suffer from the difficult task of selecting appropriate measures and indicators to monitor health services [6], while the possibility of using performance information to manage health performance is limited by dysfunctions such as opportunistic behaviour and goal ambiguity [14]. Finally, a common unintended consequence of performance measurement misuse in healthcare is associated with the increased friction between physicians and managers, which the design of such a tool can lessen by promoting horizontal communication and collaboration at different organization levels [15]. In this regard, the development of performance indicators is a powerful tool to promote organizational learning and improve the internal climate and interorganizational collaboration, as shown by the study of Zidarov et al. [16] in the field of rehabilitation in Quebec, Canada. Many studies have also viewed performance indicators as important mechanisms for carrying out benchmarking exercises in order to improve healthcare quality and efficiency [17]. In their study on US eye hospitals, de Korne et al. [18] state that the journey matters more than the destination, as benchmarking effectiveness is strongly related to dynamic conditions such as iterative and learning processes based on cooperation between clinicians and managers.

Any PMS, especially when used to carry out comparative analyses, must take due account of the characteristics of the context within which it is developed because environmental variables, such as demographic, socio-economic, geographical and epidemiological factors, as well as internal variables, such as organizational capital and resource availability, strongly affect health service delivery and outcomes [19, 20].

It has been pointed out in the literature that the support of top managers influences middle management commitment and facilitates innovation implementation [21], whereas top managers’ dominant culture differently impacts on the values, attitudes and behaviours of the members of healthcare organizations [22]. In line with these findings, there is wide agreement on the fact that hindrances to the success of performance measurement in the healthcare sector are mainly cultural and can be addressed by the legitimacy of policy-makers, the leadership of top managers and their ability to involve the whole organization [23].

In particular, the process of developing a PMS seems to make a difference and a number of studies have focused on how to cope with the methodological challenges and the key steps needed to construct and compare healthcare performance indicators [7, 24]. In this regard, Giovanelli et al. [8] showed the importance of involving practitioners and researchers from different backgrounds when designing a PMS for local public healthcare authorities (LHAs) in Italy.

While there are well-established frameworks for measuring and comparing the performance of secondary care, and especially hospital care [25], some other areas of healthcare such as primary care and prevention care have been rather neglected to date. However, some broadly used frameworks such as the WHO’s framework for action for strengthening health systems [26], whilst not specific to prevention, have represented valuable starting points for developing performance measures in different public health settings. In order to address the urgent need to improve the performance of health systems, the key purposes of the WHO’s framework were to promote common understanding of what a health system was and to define desirable attributes of an effective health system so as to propose a more integrated response. The framework highlighted the context-specific nature of health systems, which particularly affects preventive services and activities. Furthermore, a more recent document by the WHO [27], points out that the effort in developing informative systems for preventive and promotive care can lead to higher accountability, data comparability and trustworthiness of information, thus increasing the relevance of public health within health policy debates.

The strong heterogeneity that affects preventive services and activities around the world, on the one hand, makes it difficult to precisely identify the actors and organizational structures responsible for their delivery; on the other hand, it hinders the design of a common framework for their analysis. It also hinders the definition of best practices for improved performance. In some countries, hospitals have traditionally played a key role in providing services for the health of communities and for the whole nation [28]. In other countries, such as Italy, independent structures specifically devoted to providing services and enabling people to increase control over, and improve their health, have been constituted within LHAs. In any case, prevention care has gained increasing attention worldwide and a number of reforms have recently been aimed at enhancing its role in national health systems, such as the US Affordable Care Act of 2010. The main reason for such a new approach is the twofold benefit of prevention, as it is conducive to improving health and saving lives while it reduces the demand for health services and especially the burden of chronic diseases, thereby lowering the costs of the entire healthcare system [1]. This also explains why, since the advent of the financial crisis, strengthening prevention care has been a pillar of European policy in the health sector [29]. However, improvements in efficiency associated with the reinforcement of preventive services are difficult to measure. While several studies have assessed the positive relationship between prevention and health outcomes [30], the effects of prevention on costs are contradictory and require much more investigation [31]. To date, the common underutilization of preventive services, even when free [32], has led scholars to focus on the determinants of demand and the issue of equal access to prevention care [3, 4, 33]. In this regard, a number of cross-national comparative analyses on inequalities in preventive care use has been carried out [34, 35]. From a methodological perspective, the potential to compare prevention performance and disseminate best practices is hindered by the fact that although quality indicators of specific preventive services are well-established internationally, comprehensive and multidimensional frameworks to measure and evaluate preventive care activity have not yet been developed. Some factors make the task particularly challenging, such as the strong heterogeneity of services [5], the fragmentation of health plans and the difficulty in identifying the roles of different actors involved in the delivery and performance of preventive services such as primary care physicians, patients associations and schools [36, 37]. For instance, in Italy, preventive care is characterized by local peculiarities, which is also due to the organizational autonomy of the regions. Access inequality, which results in the underuse of preventive services, remains a serious concern [38]. Furthermore, evaluating prevention has been shown to be difficult due to the breadth of services included in this domain and the vagueness of the boundaries between prevention activities and health promotion [39]. In this regard, policy documents and plans, such as the recent National Prevention Plan (NPP), seem to have failed to provide adequate tools for monitoring, measuring and reporting the effectiveness and cost-effectiveness of preventive services [40].

## Preventive care in Italy

In Italy, preventive care is delivered by the Italian National Healthcare System (INHS). The INHS, which was established in 1978, is a publicly funded service that relies on citizens’ taxation. The reform of the Constitution in 2001 defined health as a regional matter, thereby creating a multi-tier system. Specifically, the central government defines the main areas and parameters of the healthcare services that should be equally guaranteed to all citizens throughout the country (‘livelli essenziali di assistenza’– LEA− were established by legislative decree No. 502/1992 and first defined in 2001), while regions are delegated to organize and deliver services in respect of these parameters. Each region has autonomy in choosing how to deliver these services and how to organize the healthcare system within its territory. Finally, at an operative level, with regard to long-term economic equilibrium, LHAs are responsible for providing health services, therapies and treatments for citizens either directly through their facilities or by buying them from accredited providers. LEAs, among others, define the activities of preventive care that focus on the following areas:

- hygiene and public health provides services to protect human health (i.e., prevention and control of infectious diseases, primary basic vaccination, prevention and control of infectious diseases in the school environment and lifestyle education);
- food control and hygiene surveillance deals with those activities aimed at contributing to guarantee food hygiene and health conditions through the inspections of food companies, points of sale, restaurants, school canteens as well as the control of water for food use;
- occupational safety and prevention refers to checks and inspections of workplaces in order to assess compliance with safety regulations and prevent and avoid accidents;
- public health and veterinary services concern various monitoring activities in livestock to prevent disease in cattle and ensure public health for both animals (in particular farmed animals) and humans;
- individual prevention care is focused on specific screening programmes to prevent some of the serious diseases, such as colorectal cancer, cervical cancer, mammary carcinoma and so on.

From a policy-making perspective, the NPP, which derives from the National Health Plan (NHP) and must be translated by regions into the Regional Prevention Plans (RPP), is the main planning tool, even though a plethora of other programmes and objectives are set out in relation to each service. Within regions, the Department of Prevention (DP) of each LHA is in charge of RPP implementation.

At national level, preventive care is steered by the Minister of Health through two main offices that are responsible, respectively, for health prevention and veterinary health (see Prime minister decree No. 59/2014). The organization of preventive care at the central level has influenced its organization at the regional level, since several Regions have adopted this categorization. Furthermore, it has also been used in the main plans. In particular, the NPP 2014–2018 identified two main areas of activities: health promotion and prevention care and veterinary and food safety. Health promotion and prevention care deals with all those activities that address human prevention and safety, particularly hygiene and public health services to protect human health, occupational safety and prevention checks and inspections in workplaces, as well as individual prevention focused on specific screening programmes. Veterinary public health and food safety is a twofold area focused on food control and hygiene surveillance on the one hand and veterinary services on the other hand.

In recent years, Italy has often occupied the top positions in world rankings of the best healthcare services. In 2017, the Bloomberg Global Health Index (which takes into account factors such as life expectancy and health risks derived from the environment and lifestyle, such as pollution, nutrition, tobacco and alcohol consumption, as well as basic vaccination coverage) identified Italy as the healthiest country. Prevention activities, among others, are supposed to have provided a significant contribution to education in relation to dietary habits, general lifestyle and vaccination levels.

Italy’s expenditure on prevention is approximately 4 % of the total public health expenditure, above the average of OECD countries during the 2012–2016 period (OECD Health Statistics, 2018), but with remarkable differences at the regional level [41].

## Methods

This work aims to identify a methodology for managing performance in preventive care. The study was carried out in Italy, where preventive care is free for the population in relation to determined LEAs and delivered by the national healthcare system. In particular, the research setting was an Italian regional healthcare system undergoing a major reorganization process.

The development of the PMS was undertaken from a constructivist perspective and used a participatory action research approach, which implies a collaborative method that is well-known in management accounting [42] and public health research [43]. It is based on the idea that the world and the organizations within it can be better understood and changed through mutual reflection and the intervention of researchers and practitioners whose interaction promotes the advancement of scientific knowledge and, at the same time, encourages the identification of innovative paths for solutions to operational problems.

The decision to use interventionist research in the present work is easily justified due to the specific characteristics of the context under investigation. The activities aimed at protecting public health and collective prevention present a high level of complexity that can be better overcome through a participatory approach. With specific reference to evaluation, it is believed that this approach can enhance the development of new indicators that are able to more accurately represent the perspectives of stakeholders or reveal neglected aspects.

The research project took 24 months to complete and was articulated in four phases. The first phase of the project set the objectives of the work and designed the research methodology. Because the region’s healthcare system was undergoing a major reorganization at the time, the PMS should have been particularly useful for: i) monitoring preventive services in order to assess and compare performance across the region; ii) planning future activities on a more informed basis; and iii) supporting the regional Ministry, which was leading the change in the reorganization of preventive care.

In line with the literature [24], the goals of the PMS were then agreed as follows: to improve the link between performance evaluation and national and regional planning, to safeguard completeness by considering all the different areas and services provided by DPs, to provide an adequate balance between efficiency and effectiveness by developing, when possible, an indicator of effectiveness and an indicator of efficiency for each service) and to safeguard information parsimony. Under the leadership of the academic research group, the research team then agreed on the methodology.

The confrontation with practitioners led to select effectiveness as a performance dimension instead of outcome. Although it is not disputed that “the best measure of a health system’s performance is its impact on health outcomes” [26], it should be noted that assessing outcome is a challenging task. In particular, it is difficult to identify a direct relationship between the service provided and its impact on health. To overcome this issue, it was decided to indirectly measure the outcome of preventive services by looking at the effectiveness of DPs. The underlying assumption is that a service provider capable of achieving objectives related to well-established standards of activity will increase the outcome of its services. In the second phase, the proposal of the academic research group was debated and refined during several meetings with the internal experts of the research team, while the third phase was devoted to defining the final version of the PMS through an external revision process, carried out in two subsequent steps.

During the final phase of the research, the PMS was experimentally applied to the regional healthcare system (seven out of eight DPs took part in the experiment) with the aim of defining the final version. The experiment, based on activity in 2015, began in June 2016 and ended in December 2016.

## Results

The results presented in this section are divided into the four phases to highlight the intermediate results and describe the complex path that enabled the achievement of the objectives of the research.

### Phase 1: the project-planning phase (June to July 2015)

This phase began with the establishment of the research team, a task carried out by the Regional Minister of Health and Social Affairs who played the key role of sponsor and coordinator throughout the project. In line with the participatory approach and taking into account the heterogeneity of the activities of DPs, the research team consisted of:

- three senior executives from the Regional Ministry of Health and Social Affairs;
- an academic research group (made up of four academics in the field of healthcare management) with previous experiences of designing PMSs;
- three DP directors from the LHAs in the region, each with a different professional background: public health, occupational security and prevention and veterinary services.

First, the academic research group conducted an extensive literature and normative review together with an analysis of the most important documents of national (e.g., NPP, National Vaccination Plan 2012–2014 and LEA), regional (e.g., RPP) and local planning (e.g., LHAs’ strategic plans). These analyses led to the identification of national and international best practices in the field and a set of well-established measures already used in the performance evaluation of preventive care. As a result, an initial list of 216 indicators was selected.

The documentary analysis brought to light some important evidence about performance evaluation in preventive health care. First, performance evaluation appeared to be more developed in some areas (e.g., veterinary and public health) and scant in others (e.g., food control and hygiene surveillance, occupational safety). Second, the analysis showed a gap in efficiency-related measures as the majority of performance indicators only focused on effectiveness or outcome. To address these issues, the research team developed a second list in which duplicate indicators were removed and further indicators for these underestimated areas and efficiency dimension were developed. The second version of the list, comprising 72 indicators, was incorporated into a preliminary proposal of the model and the indicators were grouped into three areas:

- health promotion and prevention care, which included hygiene and public health, occupational safety and prevention and individual preventive care;
- veterinary and food safety, which included veterinary services, food control and hygiene surveillance;
- DPs’ general activity, which was referred to the whole DP.

The first two areas reflect the categorization used at the national level by the Minister of Health and adopted by the NPP 2014–2018. It is worth noting that preventive care in the investigated region is managed through two distinct offices that are responsible for, respectively, health promotion and prevention care and veterinary and food safety. The creation of a specific office to lead veterinary services responded to the need to tackle the specific problems in the regional context, e.g., veterinary epidemics.

### Phase 2: the internal revision (July 2015 to March 2016)

During this phase, the team analysed and revised measures to define the first draft of the PMS, now made up of 50 indicators divided into three areas of activity and two dimensions, as shown in Table 1.

**Table 1:** The first draft of the PMS

Areas of activity	Effectiveness	Efficiency	Total
Health promotion and prevention care	14	12	26
Veterinary and food safety	17	5	22
DPs’ general activity	–	2	2
Total	31	19	50

In order to verify the measurability of the indicators, a panel of independent controllers (from the regional LHAs that were not taking part in the research) was asked to validate them. Finally, in order to verify the applicability and usefulness of the system, a pilot test based on activity in 2014 was carried out in the DPs of the directors involved in the project. To facilitate data collection and assure the homogeneity of the results, some technical devices were developed: a manual with guidelines and details about calculation formulas and sources was prepared and a spreadsheet-based tool was devised to collect information. The testing phase lasted from September 2015 to January 2016. Table 2 shows the number and the percentage of indicators calculated by all the DPs involved in the pilot test. Some significant evidence clearly emerged from these findings.

**Table 2:** Results of the pilot test

Areas of activity	Effectiveness	Efficiency	Total
Health promotion and prevention care	11 (79%)	9 (75%)	20 (77%)
Veterinary and food safety	5 (30%)	1 (20%)	6 (27%)
DPs’ general activity	–	2 (100%)	2 (100%)
Total	16 (52%)	12 (63%)	28 (56%)

First, the share of the calculated indicators was limited to 56%, thus highlighting the complexity of the process. In particular, DPs faced greater difficulties in regard to veterinary medicine and food control, while these were lesser in regard to human health promotion. In contrast, there was no remarkable difference between efficiency and effectiveness. Second, the results showed a great level of variability among the DPs, which strictly limits the comparability of the findings. This variability was due to divergent interpretations of the indicators resulting from the lack of clarity about the information provided to support data collection and the lack of homogeneity in the data sources. To overcome these issues, the research team carried out a complete revision of both the model and the supporting tools. Using a traffic-light approach [44], indicators were divided into three groups: the green group, composed of 30 indicators considered to be completely reliable and not requiring any further revision; the yellow group, composed of nine indicators requiring minor revision in order to improve their trustworthiness and homogeneity; and the red group, composed of 11 indicators that required complete revision.

### Phase 3: the external revision (March 2016 to June 2016)

Using an external revision process, the third phase allowed the final version of the PMS to be defined. In particular, it was aimed at overcoming the issues that had emerged in the pilot test and building credibility and involvement in the project by increasing transparency. Consequently, in April 2016, the PMS was presented and analysed as part of a higher post-graduate educational programme in healthcare management that involved 35 professionals from multidisciplinary backgrounds. Participants were asked to discuss the limitations of the indicators and to identify possible ways to overcome them. In June 2016, the revised system was shared and discussed with over 50 executives and professionals from all the LHAs in the regional healthcare system during a workshop held at the Regional Ministry of Health and Social Affairs. In this phase, the presence of the Regional Ministry signalled the sponsorship of the region and played a key role in improving engagement. Table 3 shows the composition of the second draft of the PMS.

**Table 3:** The second draft of the PMS

Areas of activity	Effectiveness	Efficiency	Total
Health promotion and prevention care	13 (↓1)	11 (↓1)	24 (↓2)
Veterinary and food safety	15 (↓2)	5 (=)	20 (↓2)
DPs’ general activity	–	2 (=)	2 (=)
Total	28 (↓3)	18 (↓1)	46 (↓4)

### Phase 4: the experimental application (June 2016 to June 2017)

The final phase, the experimental application in the whole regional healthcare system, enabled the development of the final version of the PMS. The lessons learnt from the pilot test suggested the following methodological devices: each DP was asked to identify a person who would be responsible for the process (usually the director of the DP); a manual with guidelines and details about calculation formulas and sources was prepared; a spreadsheet-based tool was devised to collect information; and a helpdesk was established, composed of the academic research group, the role of which was to support the internal control offices and to address the lack of homogeneity in interpreting the formulas. The academic research group elaborated the results between January and March 2017. In particular, the helpful comments provided by the LHAs’ control offices were used to enhance the clarity and trustworthiness of the formulas for the indicators. At the end of the process, 29 indicators were considered to be fully reliable, 11 indicators needed further analysis to strengthen their reliability and six indicators revealed significant issues that led to their elimination.

The results were then presented to the research team for the final revision, which led to the selection of 39 indicators, as shown in Table 4, and the elimination of seven indicators. In particular, three indicators were eliminated due to unavoidable difficulties in data collection, while a further four were temporarily excluded as these measures were considered reliable but DPs needed additional data sources to obtain trustworthy and comparable information that was not available at the time due to the limitations of their information systems.

**Table 4:** The final version of the PMS

Areas of activity	Effectiveness	Efficiency	Total
Health promotion and prevention care	12 (↓1)	9 (↓2)	21 (↓3)
Veterinary and food safety	13 (↓2)	3 (↓2)	16 (↓4)
DPs’ general activity		2 (=)	2 (=)
Total	25 (↓3)	14 (↓4)	39 (↓7)

The final step of the project was intended to complete the PMS, with the addition for each indicator of a benchmark measure and some context variables to be considered when interpreting the results (see the full list of indicators in the Additional file 1).

## Discussion

The discussion of the results focuses on the identification of critical factors in two distinct areas: general issues regarding the process of the development of a PMS in public healthcare and specific issues regarding the design of such a tool in the context of preventive care. In relation to the first set of issues, this study concurs with the concept that a continuous interactive process between practitioners and academics [8], as well as between physicians and managers within organizations [15], is crucial in designing effective performance evaluation and monitoring systems in the healthcare sector. Nevertheless, personnel involvement seems to be affected by high workloads in public health organizations, which increased in the aftermath of the recent financial crisis. Furthermore, public health personnel report an increased volume of red tape, which contributes to stripping performance measurement and evaluation systems of their real meaning. This compounds the usual scepticism surrounding performance measurement in public organizations, which is largely based on its capacity to drive the behaviour of personnel (and, consequently, organizations), towards the improvement of services and outcomes rather than merely sanctioning individuals for misconduct. This concern appears to increase when organizations go through periods of institutional change that usually trigger protection and conservation mechanisms as people under evaluation worry about losing their positions. These issues have also emerged in similar studies and can be addressed by improving transparency and communication within organizations and by enhancing professionals’ motivation to take part in the design process [18]. This study reveals that these purposes can be achieved by clearly defining roles and responsibilities among the members of the research team and finding time and ways to ensure involvement at different levels of the organization [15].

In this regard, the sponsorship of the regional government, which promoted and coordinated the project, was crucial to increasing the LHAs’ commitment and public awareness of the meaning of the process [22, 23], while the scientific leadership of the academic research group fostered credibility and authority. In turn, the group of directors of DPs involved in the project, the members of which had different specializations in the field of prevention, provided the research team with a full picture of prevention services and ensured maximum engagement within their DPs [21], which were used as a privileged field to test the system. Furthermore, discussing the system with independent panels of experts from different backgrounds in healthcare (controllers, physicians and health managers) prior to extending the proposal to practitioners, was important in terms of including multiple perspectives [11] and enhancing the objectivity of the process. Finally, the presentation of the system to all executives and professionals involved in preventive services, and its subsequent experimental application in the DPs of the region were conducive to linking theory to practice and making preventive professionals fully aware of their roles, both in the project and in the regional health reform.

The contribution of this paper can be appreciated in light of the four interconnected pillars of the WHO’s response to health systems challenges [26]. In regard to the first pillar, this study directly points to three of the six ‘building blocks’ that make up a health system: providing ‘good health services’ by finding the balance between effectiveness and minimum waste of resources, producing and analysing information on health status and performance in a reliable and timely way, and focusing, among others, on the significant prevention service of vaccines in order to ensure equitable access, coverage, quality and cost-effective use. With regard to the second pillar, that is, ‘getting results’ from health systems and programmes, the proposal of this study is in line with the ‘diagonal approach’, which is aimed at improving and extending existing interactions as well as creating better and more systematic communication by producing robust monitoring and evaluation frameworks. Finally, the findings align with the principles stated in the third and fourth pillars, which analyse the role of the WHO at country and international levels. In fact, the methodology relies on a more intense engagement of all actors involved in health sector policy and processes at country level, which is conducive to increased and shared knowledge about methods and tools, thereby promoting structured discussions and the creation of a common language for a wider variety of audiences.

In terms of the issues affecting the development of a PMS for preventive care, the main critical point that emerged from this study was the significant heterogeneity of the information sources used by the DPs, which made the experimental application of the system very difficult. First, this was due to the coexistence of traditional long-standing services, e.g., vaccinations, screenings and veterinary services and quite new services (basically those concerned with public health promotion), with the latter lacking the established and shared measures enjoyed by the former.

Second, the situation reflected the organizational heterogeneity among the DPs as they had developed their own methods and routines for the delivery of services. Practitioners complained that this had been exacerbated over the years by the proliferation of regulations and policy documents (which often refer to specific areas of prevention) at both the international and national level.

In order to comply with these directives within the appropriate time frame, DPs were compelled to develop their own solutions without consulting each other, which has resulted in different stages of development between preventive services. In addition, it was discovered that the dimension of efficiency had been almost completely disregarded. In fact, while a remarkable number of indicators of effectiveness was found in the policy and planning documents, most of the efficiency indicators had to be autonomously developed by the research team in order to measure and evaluate resource use, costs and productivity for every single service. Indeed, the current information systems proved to be rather incomplete.

These findings lead to some important implications for policy-makers. In particular, rationalizing regulations and giving health organizations and professionals the opportunity to gradually assimilate change, as well as arranging meetings to discuss new requirements and processes, are conducive to improving homogeneity in both service delivery and information sources. In this regard, collaborative methods based on the mutual reflection and intervention of academics and professionals are important for identifying and sharing best practices for measuring activities and results, thereby improving the use of information systems.

The study also highlights the need to assess the performance of health services using a small group of indicators, selected on the basis of the criteria of relevance (indicators that address health priorities), availability (indicators that can be measured with available data) and quality (indicators that capture the dimensions of performance), thus confirming the methodological indications provided by the WHO [20].

Two other general issues must be discussed. The first refers to the concept of outcome, which is preeminent in evaluating healthcare performance and should be considered in light of several environmental variables [19]. Indeed, on the one hand environmental variables play a key role in determining health outcomes, while on the other hand they cannot easily be controlled by health organizations. As a result, since health organizations can only partly influence final outcomes, it seems appropriate to primarily consider process and activity indicators when evaluating their performance. This follows the principle that the main duty of health organizations is to do the right things in the right ways in order to improve the quality of healthcare [45]. Furthermore, as environmental variables appear to be even more important for the outcomes of preventive care – since the identification of social, demographic, epidemiological and structural conditions of diverse regions strongly impact the possibility of avoiding disease – the design of a PMS for preventive care must include the contextual parameters needed to interpret the DPs’ performance and to explain differences. As the WHO indicated [20], although is fundamental to ensure an international comparison of health systems’ performance by adopting methodological indications that are internationally accepted, it is crucial that the design process of a PMS takes into account local specificities. In this regard, the International Classification of Health Intervention (ICHI) could represent a useful tool to identify and isolate preventive practices for monitoring. Nevertheless, it should be noted that the ICHI framework is often limited to a part of preventive services. For instance, with reference to Italy, it is only related to promotive and preventive health services as defined by national law (LEAs and the NPP). The identification of key environmental variables is also useful for developing standards and benchmarks to better evaluate performance within a certain region or health system [17]. Without a doubt, the relationship between process indicators and the outcomes of preventive services needs more empirical scrutiny [13].

The second issue deals with the transverse nature of preventive care, which has been enhanced by recent regulations and policies. In this case, the principle is that prevention outcomes are increasingly influenced by teamwork and, in general, by the involvement of a plethora of actors who educate and support people to safeguard their own health at different times in their lives. This is directly visible, for instance, in the area of health promotion, in which programmes usually highlight the role of schools, police and fire departments in promoting dietary habits as well as road and occupational safety. However, it is still very difficult to isolate and measure the contribution of each of the actors involved in service delivery, as well as their mutual relationships, because information systems have not yet evolved towards a network approach and still seem to be mainly oriented around the internal elements of each organization.

The limitations of this study are noted in the suggestions for further research. First, the research was carried out in the preventive care services of the INHS, which has its own specific institutional and organizational characteristics, and a regional health system, which was undergoing a thorough reform process, was chosen as the field of study. There is an incentive to extend the application of the methodology elsewhere in order to test its general principles and flexibility, that is, to test how it would need to be adapted in relation to the peculiarities of healthcare systems and the preventive care services within them. Furthermore, for final validation, it will also be important to test the methodology in health contexts that are characterized by different stages of development and different problems, since the health needs of individuals and groups and the risk factors in society continually evolve over time. For example, it would be useful to carry out such an assessment in countries that are dealing with the issues related to the health of migrants and refugees and with environmental protection. Finally, following a similar participatory action research approach, future research will aim to investigate the critical factors that are associated with the phases that follow the design of a PMS, that is, its actual implementation and use within health organizations.

## Conclusions

Notwithstanding the increased attention on prevention care in health policy reforms around the world due to its combined effect of improving public health and reducing the costs of health systems, decision-makers continue to suffer from a lack of information and managerial tools to effectively plan and control the organization and delivery of preventive services. This study aimed to define a methodology to design a PMS in order to support decision-making in such a specific area of healthcare. The complexity, heterogeneity and, in some cases, the innovativeness of preventive services require the identification of critical factors that can influence the design and implementation of advanced managerial systems for the improvement of the quality of this kind of service. The findings of this research provide insight into the peculiarities that affect preventive activities and constitute a baseline for addressing the methodological issues that arise when assessing the performance of such a poorly considered area of healthcare. Furthermore, this study, which was conducted in an Italian regional healthcare system that was undergoing a major reorganization, underpins the implications and recommendations addressed to an international audience in keeping with the idea that countries have much to learn from sharing solutions and best practices.

The critical factors that affect the design of a PMS in preventive care can be placed in two categories: general issues regarding the process of development of a PMS in healthcare and specific issues regarding the design of such a system in preventive care.

Among the critical factors of the first category, the study identified:

- the high workloads and the volume of red tape that affect working conditions in public health organizations;
- the effect of the financial crisis and institutional changes within an organization on the involvement of health professionals;
- transparency and open communication during the process of development.

Then, among the critical factors of the second category the study highlighted:

- the significant heterogeneity of the information sources;
- organizational heterogeneity, in terms of methods and routines for the delivery of preventive services;
- the proliferation of regulations and policy documents;
- the lack of consideration of the dimension of efficiency.

The main implications for policy-makers are that regulations should be rationalized and reforms should be gradually implemented within public health organizations, while collaborative methods that involve academics, professionals and policy-makers should be used to design PMSs in healthcare, and especially in preventive care. The participatory approach improves the identification and sharing of best practices for measuring activities and results and enhances transparency and involvement. Nevertheless, it should be accompanied by a clear definition of roles and responsibilities among the different participants, who should be involved at appropriate times and in appropriate ways.

## Additional file

Additional file 1: Performance management system for the preventive healthcare**,** This table shows the final version of the performance management system composed of 39 indicators divided into three areas of activity and two dimensions (DOCX 13 kb)

## Abbreviations

DP: Department of Prevention
INHS: Italian National Healthcare System
LEA: Livelli Essenziali di Assistenza (Essential Level of Care)
LHA: Local Healthcare Authority
NPP: National Prevention Plan
PMS: Performance Management System
RPP: Regional Prevention Plan
