# Supplementary material for: A methodology to design a performance management system in preventive care
Source: BMC Health Serv Res. 2018 Dec 29;18:1002. doi: 10.1186/s12913-018-3837-8 (PMC6311075; doi:10.1186/s12913-018-3837-8)
Supplement: Supplementary file 1 — Performance management system for the preventive healthcare, This table shows the final version of the performance management system composed of 39 indicators divided into three areas of activity and two dimensions (DOCX 13 kb) [file 12913_2018_3837_MOESM1_ESM.docx]

**Additional file 1**

**Table S5 ‒** Performance management system for the preventive healthcare

| **Health promotion and preventive care ‒ Effectiveness** |
| --- |
| MMR vaccination coverage of children aged 24 months |
| Flu vaccination coverage |
| Percentage of people participating in a 1st level screening programme |
| Level of coverage of mammography screening |
| Level of coverage of colorectal cancer screening |
| Percentage of construction companies inspected (including asbestos-related) on target |
| Percentage of farms inspected on target |
| Percentage of company inspections (excluding construction companies and farms) on target |
| Percentage of asbestos reclamation projects examined |
| Percentage of occupational accident enquiries completed |
| Percentage of occupational disease enquiries completed |
| No. of learning programmes for external users organized by the occupational safety and prevention office |
| **Health promotion and preventive care ‒ Efficiency** |
| Unit cost of flu vaccination |
| Unit cost of hygiene and public health |
| Unit cost of mammography screening programme |
| Unit cost of colorectal screening programme |
| Construction companies inspections' productivity |
| Asbestos reclamations' productivity |
| Occupational accident enquiries' productivity |
| Occupational disease enquiries' productivity |
| Learning programmes' productivity |
| **Veterinary and food safety ‒ Effectiveness** |
| Level of coverage of bovine tuberculosis monitoring |
| Level of coverage of swine fever monitoring |
| Bluetongue vaccination coverage |
| Level of coverage of animal registration monitoring |
| Level of coverage of animal welfare monitoring |
| Level of coverage of food contamination monitoring |
| Level of coverage of butchers monitoring |
| Level of coverage of dairies monitoring |
| Level of coverage of food factories monitoring |
| Level of coverage of mineral water, spring water and food water monitoring |
| Level of coverage of water for human consumption monitoring |
| Level of coverage of school and workplace canteens monitoring |
| Level of coverage of phytosanitary products monitoring |
| **Veterinary and food safety ‒ Efficiency** |
| Veterinary medicine workload (measured in livestock units) |
| Veterinary medicine workload (measured in number of farms) |
| Food control and hygiene surveillance workload |
| **DPs' general activity ‒ Efficiency** |
| DPs' general activity per capita cost |
| DPs' employee per capita cost |
